# Gender differences in health-related quality of life in people with severe mental illness

**DOI:** 10.1371/journal.pone.0229236

**Published:** 2020-02-21

**Authors:** Ester Colillas-Malet, Gemma Prat, Albert Espelt, Dolors Juvinyà

**Affiliations:** 1 Facultat de Ciències de la Salut de Manresa, Universitat de Vic–Universitat Central de Catalunya (UVic-UCC), Av. Universitària, Spain; 2 Grup SaMIS (Salut Mental i Innovació Social), Divisió de Salut Mental de la Fundació Althaia, Manresa, Spain; 3 CIBER de Epidemiología y Salud Pública, Instituto de Salud Carlos III, C/ Melchor Fernández Almagro 3–5, Spain; 4 Departament de Psicobiologia i Metodologia en Ciències de la Salut, Universitat Autònoma de Barcelona (UAB), Cerdanyola del Vallès, Spain; 5 Grup de recerca de salut i atenció sanitària de la Universitat de Girona, Girona, Spain; University of Sao Paulo Medical School, BRAZIL

## Abstract

**Introduction and purpose:**

The purpose was to analyze socioeconomic and clinical factors of psychosocial functioning and self-perception in relation to health-related quality of life (HRQOL) in people with severe mental health illness (SMI) by gender.

**Materials and method:**

A cross-sectional study was conducted on a sample of 133 women and 90 men. Recorded variables: HRQOL, SF-36 Physical Component Scores (PCS) and Mental Component Scores (MCS); sociodemographic and clinical data on psychosocial and self-perception functioning. Correlational studies using raw and adjusted linear regression models to evaluate the factors associated with HRQOL by obtaining coefficients, p-values and respective confidence intervals.

**Results:**

The mean PCS for women and men was 44.6 and 49.0 (p = 0.004) and 36.4 and 37.5 (p = 0.575), respectively for MCS. The factors associated with PCS in women were age, -0.2(-0.4:0); in work, 4.2(0.3:8.2); with an income higher than 700 euros/month, 4.4(1:7.7). In men, these factors were education level, 6.1(0.4:11.7); belief that they would not need help in the future, 4.6(0.1:9.2) and a higher need for psychosocial services, -6.6(-11.1:-2). Factors associated with MCS in women were, in work, 6.1(1.5:10.7); and having a high number of friends, 6.6(2.1:11.1). In men, these factors were, living alone, -7.1(-12.7:-1.4); lack of economic benefits, 8.5(3.2:13.8); and a higher need for psychosocial and social services, -3.6(-7.1:-0.2) and -7.7(-13.4:-2).

**Conclusions:**

The dimensions affected and the factors that are associated with HRQOL for people with SMI differ by gender. Therefore, these differences should to be taken into account when designing interventions for improving HRQOL.

## Introduction

One of the main objectives of the work carried out by national[1] and international[2] health organizations is the improvement of health-related quality of life (HRQOL). HRQOL is defined as the level of well-being based on an individual’s evaluation of how various aspects of their life (physical, psychological, social and spiritual well-being) impact their health status[3]. There are many people with mental health problems in the community and their HRQOL is especially important to consider[4]. They often live in more disadvantaged conditions compared to the rest of the general population[5]. These conditions can lead to economic, social and emotional problems. In some cases, these people show dissatisfaction with their social relationships, economic conditions and personal safety[6,7]. The poorest aspects of HRQOL for people with severe mental illness (SMI) are social activity followed by role-emotional limitations and mental health[8].

Women present worse HRQOL than men in the general population[9,10] as well as among people with SMI. At the same time, individuals with SMI present lower HRQOL scores than the general population, as seen in a study that showed persons with obsessive-compulsive disorder and schizophrenia having lower HRQOL scores than the Spanish population, especially in the areas of mental health[8]. This gender relationship is because this factor interacts in all of the social determinants of health as a structural determinant[11] and, at the same time, as an axis of inequality[12]. In fact, it has been seen that gender role conflicts, overall work load and unpaid work have adverse effects on women’s quality of life[13].

Despite a lack of consensus on the influence of socioeconomic factors and HRQOL on people with SMI[14], it has been observed that factors associated with better HRQOL are being male[15–17], young[18–20], working[21–23], having higher income[23,24], living with the family[25] and having a social network (size[26] and satisfaction[22]). Comorbidity[27,28], as well as factors such as symptoms of anxiety and depression[22,29,30] and their severity, are negatively associated with HRQOL[23]. Self-referenced psychosocial needs are also negatively associated with HRQOL in terms of number and typology[22,31–33].

Given that there is no data showing how factors differentially affect the HRQOL of women and men with SMI, the purpose of this study is to analyze, from a gender perspective, which sociodemographic, clinical and psychosocial functioning factors, just as self-perception of the current and future effects of the illness factors are associated with the health-related quality of life of people with severe mental illness.

## Materials and methods

### Design and study population

#### Cross-sectional study

The study population was composed of people diagnosed with SMI undergoing ambulatory monitoring in 2009 at the mental health center of the Mental Health Division of the Fundació Althaia in Manresa, an organization serving a population of 215,000.

### Sample selection

There was a total of 783 people who met the inclusion criteria. The inclusion criteria were: between 18 and 65 years old; having received ambulatory treatment at the mental health center; complied with one of the diagnostic criterion of the International Statistical Classifications of Diseases and Related Health Problems, 10a. Revision (ICD-10), for the diagnosis of SMI (schizophrenia and other psychotic illnesses, affective disorders such as major recurrent depression and bipolar disorders I and II, anxiety disorders such as obsessive-compulsive disorder with or without agoraphobia, and finally, borderline personality disorder); and willingness to take part in the study. The sample was selected by simple random sampling and the size was determined based on the known size of the population that met inclusion criteria, with a 5% error and a 3% accuracy. The theoretical sample was 258 people diagnosed with SMI who carried out ambulatory follow-up at mental health center. The final participation was 86.4% (223 people), 133 women (59.6%) and 90 men (40.4%). All the participants signed an informed consent form at the beginning of the assessment and the study was approved by the clinical research ethics committee of the Unió Catalana d’Hospitals.

The participants were recruited by telephone, which also served to specify the date and time for the interview. The interview consisted of a 30-minute evaluation where a trained interviewer filled out the various questionnaires.

### Variables

#### Dependent variables

Two measurements associated with HRQOL were taken, the Physical Component Score (PCS) and the Mental Component Score (MCS). These two variables were calculated using the Spanish version of the Medical Outcome Study Short Form 36 *(SF-36)* [34] called *Cuestionario de Salud SF-36* [35]. It is a questionnaire consisting of 36 questions that measure subjective quality of life by addressing aspects of the person’s daily life and identifying both the positive and negative states of PCS and MCS. These two components are divided into 8 dimensions: physical functioning, social functioning, physical role limitations, mental health role limitations, vitality, bodily pain and perceptions of general health. The scores range from 1 to 100 for both components, with 100 being a score that indicates optimal health and 0 showing a poor state of health. The mean of the general population is 50 (SD = 10) and higher or lower values are interpreted as better or worse, respectively, with respect to the reference population.

#### Independent variables

Sociodemographic, clinical, psychosocial functioning and self-perception variables were measured. We administered our own questionnaire relative to sociodemographic and clinical variables. Information was collected on: gender, age, civil status, number of friends, work status, level of education, living arrangement, socioeconomic status, clinical diagnosis, (axis I and III of ICD-10), comorbidity, etc. An evaluation questionnaire regarding psychosocial functioning of psychosocial needs was also administered. Specifically, this was the Spanish version of the Camberwell Assessment of Need (CAN-R)[36] called *Cuestionario Camberwell para la evaluación de necesidades*[37], which contains 88 items divided into 22 areas or psychosocial needs: accommodation; food; looking after the home; self-care; daily activities; physical health; psychotic symptoms; information on condition and treatment; psychological distress; safety to self; safety to others; alcohol, drugs; company; intimate relationships; sexual expression; care of offspring; primary education; telephone; transport; money; and economic benefits. All the areas are scored from 0 to 1 with: 0, no problem; 1, moderate or serious problem due to help given. The scoring of the 22 areas is grouped into 5 dimensions: Basic Needs (3 items); Health (7 items); Social (3 items); Functioning (5 items); and Services (4 items) as found in other studies [38,39]. Finally, we administered our own questionnaire to collect information on current and future effects of the participant’s illness, as for example, if the SMI had had consequences on their social life and leisure activities, if these social consequences had improved or worsened their social relationships, if they had considered what possibilities would be in the future and if they thought that would need help.

### Analysis of the data

All data were analyzed separately for women and men[40]. A univariate descriptive analysis was performed of the variables for the purposes of describing the sociodemographic, clinical, psychosocial, current and future self-perception profiles of people with SMI included in the sample. A frequencies and percentages analyses were performed to show the distribution of the qualitative variables. A study of the means and measurements study of central tendency was undertaken for the quantitative variables. To examine the different patterns between women and men, the Chi-squared (X^2^) test was done for the qualitative variables and the Student’s T Test for the quantitative variables. To ascertain the different scoring of PCS and MCS of HRQOL, the mean scoring of each variable (PCS and MCS) was calculated with their confidence intervals at 95% for each independent variable. The variables that presented a p<0.1 in the bivariate model were included in the multivariate models. In addition, the colineality of these variables was studied. Finally, to know which variables were associated with PCS and MCS, raw and adjusted linear regression models were estimated by obtaining the coefficients for the respective p-values and confidence intervals. The normality of the linear regression model residuals was checked, and they followed a normal distribution (p>0.01).

All the statistical analyses were performed with the Stata 15 statistical software package.

## Results

No statistically significant differences by gender were observed in the distribution of characteristics of the sample except: civil status (48.9% women were married as opposed to 35.6% of men, p = 0.05); age (${\bar{x}}_{women}$ = 44.9 years and ${\bar{x}}_{men}$ = 41.3 years; p = 0.01); number of children (${\bar{x}}_{women}$ = 1.4 and ${\bar{x}}_{men}$ = 0.7; p = 0.00); and self-perceived psychosocial services needs (${\bar{x}}_{women}$ = 0.3 and ${\bar{x}}_{men}$ = 0.1; p = 0.02). In addition, approximately 30% of the participants were in work. The most common clinical diagnosis among women was depressive disorder and bipolar disorder I and II (34.6% for both). The most common among men was schizophrenia and bipolar disorder (30.0% and 28.9%, respectively) in addition to 60% presenting physical comorbidity. Between 40% and 50% perceived a worsening of social relations and leisure activities due to SMI (Table 1).

**Table 1:** Characteristics by gender.

		WOMEN		MEN		sig.
		n	%	n	%	
**Civil Status**	Married	65	48.9	32	35.6	0.05
** **	Not married (single, separated, widow)	68	51.1	58	64.4	
**Education level**	Primary education	72	54.1	37	41.1	0.07
	Secondary education	43	32.3	43	47.8	
** **	University studies	18	13.5	10	11.1	
**Work Status**	Employed	35	26.3	27	30.0	0.55
	Unemployed (+students and paid sick leave)	98	73.7	63	70.0	
**Living situation**	Alone	18	13.5	18	20.0	0.20
** **	With someone	115	86.5	72	80.0	
**Number of friends**	0–3	51	38.4	29	32.2	0.62
	4–6	37	27.8	29	32.2	
	≥7	45	33.8	32	35.6	
**Legal status**	Not disabled	92	69.2	69	76.7	0.22
	Disabled	41	30.8	21	23.3	
**Degree of impairment**	<33%	94	70.7	59	65.6	0.42
	>33%	39	29.3	31	34.4	
**Degree of disability**	Yes	60	45.1	35	38.9	0.36
	No	73	54.9	55	61.1	
**Economic benefits**	Receives benefits	96	72.2	66	73.3	0.85
	Does not receive benefits	37	27.8	24	26.7	
**Monthly income**	<700	49	36.8	31	34.4	0.71
	>700	84	63.2	59	65.6	
**Clinical diagnosis (Axis I and II)**^**a**^	Schizophrenia and other psychotic disorders	25	18.8	27	30.0	0.07
	Depression disorder	46	34.6	21	23.3	
	Bipolar disorder I and II	46	34.6	26	28.9	
	Others	16	12.0	16	17.8	
**Comorbidity (Axis III)**^**a**^	No Comorbidity	42	31.6	34	37.8	0.34
	Comorbidity	91	68.4	56	62.2	
**Social consequences and leisure activities**	Yes	100	75.2	60	66.7	0.17
	No	33	24.8	30	33.3	
**Social relationships**^**b**^	Improved or no change	53	39.9	41	45.6	0.28
	Worse	72	54.1	40	44.4	
	DK/NA	8	6.0	9	10.0	
**Possibilities for the future**	Yes	62	46.6	48	53.3	0.33
** **	No	71	53.4	42	46.7	
**Help in the future**	Yes	72	54.1	47	52.2	0.78
	No	28	21.1	17	18.9	
	DK/NA	33	24.8	26	28.9	
		**M**	**SD**	**M**	**SD**	**sig.**
**Age**		44.9	9.8	41.3	10.7	0.01
**Num. of children**		1.4	1.3	0.7	0.9	0.00
**Num. of needs CAN**^**c**^		3.0	1.9	2.6	1.6	0.20
**Type of needs**^**c**^	Basic	0.0	0.2	0.0	0.0	0.06
	Social	0.4	0.7	0.5	0.7	0.59
	Functioning	0.3	0.6	0.3	0.6	0.91
	Health	1.9	1.0	1.7	0.9	0.11
	Services	0.3	0.5	0.1	0.4	0.02
**SF-36**^**d**^	Physical Component Score	44.6	11.5	49.0	9.8	0.00
	Mental Component Score	36.4	13.9	37.5	13.5	0.58

M: Mean; SD: Standard Deviation; DK: Does not know; NA: No answer

^a.^ According to Manual DSM-IV

^b.^ Social relationships and activities perceptions

^c.^ According the Camberwell Assessment of Need Questionnaire (CAN-R)

^d.^ Quality of Life Questionnaire SF-36

The PCS for HRQOL shows lower scores in women with SMI than men with SMI (${\bar{x}}_{women}$ = 44.6 and ${\bar{x}}_{men}$ = 49.0; p = 0.00) (Table 1). The women presenting better PCS were: unmarried, $\bar{x}$ = 46.3; had higher levels of education/college educated, $\bar{x}$ = 51.7; in work, $\bar{x}$ = 52.3; had bipolar disorder, $\bar{x}$ = 49.4; without physical comorbidity, $\bar{x}$ = 49.9; and had seen their social relationships and leisure activities as having improved or not changed, $\bar{x}$ = 49.0. However, age (r = -0.3) and had a lower number of psychosocial needs (r = -0.5) was negatively associated with PCS in women. On the other hand, men, like women, who presented better PCS were: in work, $\bar{x}$ = 52.2; without physical comorbidity, $\bar{x}$ = 51.6; and had seen their social relationships and leisure activities as having improved or not changed, $\bar{x}$ = 52.4. However, age (r = -0.2) and had a lower number of psychosocial needs (r = -0.5) also was negatively associated with PCS in men (Table 2).

**Table 2:** Physical Component Score (PCS) of HRQOL by Gender.

		WOMEN		MEN	
		M (IC95%)	sig.	M (IC95%)	sig.
**Civil Status**	Married	42.8 (40:49.1)	0.08	48.6 (45.1:52.1)	0.82
** **	No married (single, separated, widow)	46.3 (43.5:49.1)		49.1 (46.5:51.7)	
**Education level**	Primary education	41.8 (39.3:44.2)	0.00	48.1 (44.7:51.5)	0.17
	Secondary education	46.4 (42.8:50)		48.4 (45.5:51.4)	
** **	University studies	51.7 (46.6:56.8)		54.4 (51.1:57.7)	
**Work Status**	Employed	52.3 (49.5:55.1)	0.00	52.2 (48.6:55.8)	0.04
	Unemployed (+students and paid sick leave)	41.9 (39.6:44.1)		47.6 (45.1:50.1)	
**Living situation**	Alone	46.9 (40.7:53.2)	0.36	47.1 (40.8:53.3)	0.37
	With someone	44.2 (42.1:46.3)		49.4 (47.3:51.6)	
**Number of friends**	0–3	43.7 (40.6:46.8)	0.66	48.8 (45.5:52.1)	0.94
	4–6	44.4 (40.2:48.6)		49.5 (45.4:53.5)	
** **	≥7	45.8 (42.6:49)		48.6 (45.3:52)	
**Legal status**	Not disabled	45.6 (43.1:48.1)	0.13	49.4 (47:51.7)	0.48
	Disabled	42.4 (39.3:45.4)		47.6 (42.9:52.3)	
**Degree of impairment**	<33%	46.2 (43.9:48.6)	0.01	48.8 (46.4:51.2)	0.84
	>33%	40.7 (37.2:44.3)		49.2 (45.2:53.3)	
**Degree of disability**	Yes	41 (38.3:43.7)	0.00	47.9 (44.2:51.6)	0.42
	No	47.6 (44.9:50.3)		49.6 (47.2:52.1)	
**Economic benefits**	Receives benefits	42.1 (39.8:44.4)	0.00	47.9 (45.5:50.4)	0.10
	Does not receive benefits	51.1 (48:54.2)		51.8 (48:55.6)	
**Monthly income**	<700	40.4 (37.4:43.4)	0.00	47.5 (43.8:51.2)	0.31
	>700	47.1 (44.6:49.6)		49.7 (47.2:52.2)	
**Clinical diagnosis (Axis I and II)**^**a**^	Schizophrenia and other psychotic disorders	45.3 (40.3:50.2)	0.00	49.5 (45.9:53.2)	0.35
	Depression disorder	40.5 (37.3:43.7)		45.6 (40.6:50.6)	
	Bipolar disorder I and II	49.4 (46.4:52.5)		50.4 (47.1:53.6)	
** **	Others	41.5 (36.7:46.3)		50.1 (45.4:54.8)	
**Comorbidity (Axis III)**^**a**^	No Comorbidity	49.9 (46.5:53.2)	0.00	51.6 (48.9:54.3)	0.04
	Comorbidity	42.2 (39.9:44.5)		47.3 (44.5:50.2)	
**Social consequences and leisure activities**	Yes	42.8 (40.5:45)	0.00	47.6 (44.9:50.4)	0.07
	No	50.2 (46.5:53.9)		51.6 (48.8:54.4)	
**Social relationships**^**b**^	Improved or no change	49 (46.3:51.6)	0.00	52.4 (49.8:55.1)	0.01
	Worse	41.5 (38.7:44.2)		45.8 (42.6:49)	
	DK/NA	43.9 (36:51.9)		47.2 (41.5:53)	
**Possibilities for the future**	Yes	47.5 (44.7:50.3)	0.01	50.1 (47.3:52.9)	0.25
	No	42.1 (39.4:44.8)		47.7 (44.6:50.8)	
**Help in the future**	Yes	41.5 (38.8:44.3)	0.00	46.3 (43.1:49.5)	0.01
	No	49.3 (45.1:53.5)		54.6 (51.6:57.5)	
	DK/NA	47.4 (44.2:50.6)		50.1 (47.2:53)	
		**r (IC95%)**	**sig.**	**r (IC95%)**	**sig.**
**Age**		-0.3 (-0.2:-0.4)	0.00	-0.2 (-0.1:-0.3)	0.06
**Num. of children**		-0.1 (-0.1:-0.2)	0.10	-0.2 (-0.1:-0.3)	0.09
**Num. of needs CAN**^**c**^		-0.5 (-0.4:-0.6)	0.00	-0.5 (-0.4:-0.6)	0.00
**Type of needs**^**c**^	Basic	-0.2 (-0.1:-0.3)	0.03	-	-
	Social	-0.1 (0:-0.2)	0.32	-0.2 (-0.1:-0.3)	0.03
	Functioning	-0.3 (-0.2:-0.4)	0.00	-0.2 (-0.1:-0.3)	0.04
	Health	-0.6 (-0.5:-0.6)	0.00	-0.5 (-0.4:-0.6)	0.00
	Services	-0.2 (-0.1:-0.3)	0.04	-0.2 (-0.1:-0.3)	0.08

M: Mean; IC95%: Confidence Interval of 95%; r: Linear regression coefficient; DK: Does not know; NA: No answer

^a.^ According to Manual DSM-IV

^b.^ Social relationships and activities perceptions

^c.^ According the Camberwell Assessment of Need Questionnaire (CAN-R)

The variables associated with PCS in women, explaining 41.4% of the variance, were: age [β_a_ = -0.2 (95% CI: -0.4;0)]; in work [β_a_ = 4.2 (95% CI: 0.3;8.2)]; had income of greater than 700 euros/month [β_a_ = 4.4 (95% CI: 1;7.7)]; had a high number of health-related needs [β_a_ = -4.4 (95% CI: -6.2;-2.6)]; and did not have comorbidity [β_a_ = 4.5 (95% CI: 1.1;7.9)]. These last two are also associated with men’s PCS in addition to having a higher level of education [β_a_ = 6.1 (95% CI: 0.4; 11.7)]; belief that they would not need assistance in the future [β_a_ = 4.6 (95% CI: 0.1;9.2)]; and having a higher number of needs in the area of services [β_a_ = -6.6 (95% CI: -11.1;-2.0)]. All of these explain 38.6% of the variance in men. Clinical diagnosis is not associated with PCS in either women or men (Table 3).

**Table 3:** Model of the Physical Component Score of HRQOL by Gender.

**WOMEN**		**Coefficient (IC95%)**	**sig.**	**Adjusted coefficient (IC95%)**	**sig.**
**Work Status**	Unemployed	0		0	
	Employed	10.4 (6.3:14.6)	0.00	4.2 (0.3:8.2)	0.03
**Monthly income**	<700	0		0	
	>700	6.7 (2.8:10.6)	0.00	4.4 (1:7.7)	0.01
**Comorbidity (Axis III)**^**a**^	Comorbidity	0		0	
	No Comorbidity	7.7 (3.6:11.7)	0.00	4.5 (1.1:7.9)	0.01
**Age**		-0.4 (-0.6:-0.2)	0.00	-0.2 (-0.4:0)	0.01
**Type of needs**^**b**^	Health	-6.5 (-8.2:-4.8)	0.00	-4.4 (-6.2:-2.6)	0.00
**MEN**		**Coefficient (IC95%)**	**sig.**	**Adjusted coefficient (IC95%)**	**sig.**
**Education level**	Primary education	0		0	
	Secondary	0.3 (-4:4.6)	0.89	-3 (-6.6:0.6)	0.11
	University	6.3 (-0.6:13.2)	0.07	6.1 (0.4:11.7)	0.04
**Comorbidity (Axis III)**^**a**^	Comorbidity	0		0	
	No Comorbidity	4.3 (0.1:8.4)	0.04	4.2 (0.7:7.7)	0.02
**Help in the future**	Yes	0		0	
	No	8.3 (3:13.5)	0.00	4.6 (0.1:9.2)	0.05
**Type of needs**^**b**^	Health	-5.3 (-7.4:-3.3)	0.00	-5.6 (-7.7:-3.6)	0.00
	Services	-4.9 (-10.3:0.6)	0.08	-6.6 (-11.1:-2)	0.01

IC95%: Confidence Interval of 95%

^a.^ According to Manual DSM-IV

^b.^ According the Camberwell Assessment of Need Questionnaire (CAN-R)

Men and women had similar MCS scores for HRQOL (${\bar{x}}_{women}$ = 36.4 and ${\bar{x}}_{men}$ = 37.5) (Table 1). The women who had better MCS were those who: had secondary education, $\bar{x}$ = 40.9; were in work, $\bar{x}$ = 44.9; had more than 7 friends, $\bar{x}$ = 41.4; had bipolar disorder, $\bar{x}$ = 41.1; and those that perceived that their social relationships and leisure activities had improved or not changed, $\bar{x}$ = 43.9. However, had a lower number of psychosocial needs (r = -0.5) was negatively associated with MCS in women. For their part, men who presented better MCS, like their female counterparts, were: in work, $\bar{x}$ = 45.9; and had more than 7 friends, $\bar{x}$ = 42.7. In addition, unlike women, men living with someone presented better MCS, $\bar{x}$ = 39.0. However, had a lower number of psychosocial needs (r = -0.4) also was negatively associated with MCS in men (Table 4).

**Table 4:** Mental Component Score (MCS) of HRQOL by gender.

		WOMEN		MEN	
		M (IC95%)	sig.	M (IC95%)	sig.
**Civil Status**	Married	35.9 (32.3:39.5)	0.65	37 (31.8:42.3)	0.81
** **	No married (single, separated, widow)	37 (33.8:40.2)		37.8 (34.3:41.2)	
**Education level**	Primary education	32.7 (29.7:35.8)	0.00	35.6 (31.6:39.6)	0.12
	Secondary education	40.9 (36.7:45.1)		40.4 (36.1:44.6)	
** **	University studies	40.6 (34.7:46.5)		32.2 (23.7:40.7)	
**Work Status**	Employed	44.9 (40.7:49)	0.00	45.9 (42:49.8)	0.00
	Unemployed (+students and paid sick leave)	33.4 (30.8:36.1)		33.9 (30.5:37.2)	
**Living situation**	Alone	37.5 (31.3:43.6)	0.74	31.5 (26:37)	0.03
	With someone	36.3 (33.7:38.9)		39 (35.8:42.2)	
**Number of friends**	0–3	31.4 (27.8:35)	0.00	33.2 (28.8:37.6)	0.02
	4–6	37.4 (32.9:41.9)		36.1 (31.4:40.8)	
** **	≥7	41.4 (37.5:45.3)		42.7 (37.7:47.6)	
**Legal status**	Not disabled	37.8 (34.9:40.8)	0.09	39 (35.7:42.3)	0.05
	Disabled	33.4 (29.3:37.4)		32.5 (27.2:37.8)	
**Degree of impairment**	<33%	36.4 (33.4:39.4)	0.95	38 (34.4:41.6)	0.66
	>33%	36.6 (32.6:40.5)		36.6 (31.9:41.4)	
**Degree of disability**	Yes	32.3 (28.8:35.7)	0.00	35.3 (30.9:39.6)	0.22
	No	39.9 (36.8:43)		38.9 (35.1:42.7)	
**Economic benefits**	Receives benefits	34 (31.2:36.7)	0.00	34.1 (30.9:37.3)	0.00
	Does not receive benefits	42.9 (38.8:47)		46.8 (42.5:51)	
**Monthly income**	<700	34.9 (30.9:38.9)	0.33	36 (31.2:40.7)	0.43
	>700	37.3 (34.3:40.3)		38.3 (34.7:41.9)	
**Clinical diagnosis (Axis I and II)**^**a**^	Schizophrenia and other psychotic disorders	38.9 (33.8:44)	0.00	39.2 (34.3:44.2)	0.45
	Depression disorder	33.1 (29.6:36.6)		36.2 (29.5:43)	
	Bipolar disorder I and II	41.1 (37.2:44.9)		39.3 (34.3:44.4)	
** **	Others	28.9 (20.4:37.5)		33.3 (27:39.5)	
**Comorbidity (Axis III)**^**a**^	No Comorbidity	39.1 (34.4:43.8)	0.14	35.8 (30.9:40.7)	0.35
	Comorbidity	35.2 (32.5:38)		38.5 (35:42.1)	
**Social consequences and leisure activities**	Yes	33.6 (31:36.3)	0.00	34.3 (30.9:37.7)	0.00
	No	45 (40.8:49.3)		43.9 (39.5:48.4)	
**Social relationships**^**b**^	Improved or no change	43.9 (40.4:47.4)	0.00	43.7 (39.9:47.4)	0.00
	Worse	30.5 (27.7:33.3)		32.8 (28.9:36.8)	
	DK/NA	40.7 (33:48.3)		30.1 (22:38.2)	
**Possibilities for the future **	Yes	38.4 (34.8:42)	0.13	37.1 (32.9:41.3)	0.77
	No	34.8 (31.6:38)		38 (34.1:41.8)	
**Help in the future**	Yes	31.9 (29:34.7)	0.00	33.8 (30.3:37.4)	0.00
	No	46.2 (41.1:51.3)		48 (42:54)	
	DK/NA	38.1 (33.6:42.7)		37.2 (32.1:42.4)	
		**r (IC95%)**	**sig.**	**r (IC95%)**	**sig.**
**Age**		0 (0:0.1)	0.76	-0.1 (0:-0.1)	0.61
**Num. of children**		-0.1 (-0.1:-0.2)	0.13	0 (0:0)	0.97
**Num. of needs CAN**^**c**^		-0.5 (-0.4:-0.6)	0.00	-0.4 (-0.3:-0.5)	0.00
**Type of needs**^**c**^	Basic	-0.2 (-0.1:-0.3)	0.02	-	-
	Social	-0.2 (-0.2:-0.3)	0.01	-0.3 (-0.2:-0.4)	0.01
	Functioning	-0.3 (-0.3:-0.4)	0.00	-0.2 (-0.1:-0.3)	0.11
	Health	-0.5 (-0.4:-0.5)	0.00	-0.4 (-0.3:-0.5)	0.00
	Services	-0.1 (-0.1:-0.2)	0.20	0.1 (0:0.1)	0.55

M: Mean; IC95%: Confidence Interval of 95%; r: Linear regression coefficient; DK: Does not know; NA: No answer

^a.^ According to Manual DSM-IV

^b.^ Social relationships and activities perceptions

^c.^ According the Camberwell Assessment of Need Questionnaire (CAN-R)

The variables associated with MCS that explained 38% of variance were: in work [β_a_ = 6.1 (95% CI: 1.5;10.7)]; having a large number of friends [β_a_ = 6.6(95% CI: 2.1;11.1)]; having a high number of psychosocial needs in the health area [β_a_ = -3.9 (95% CI: -6;-1.8)]; and those perceiving that their social relationships and leisure activities had improved or not changed [β_a_ = 8.6 (95% CI: 4.4;12.8)]. The last two were also associated with MCS in men in addition to living alone [β_a_ = -7.1 (95% CI: -12.7;-1.4)]; lacking economic benefits [β_a_ = 8.5 (95% CI: 3.2; 13.8)]; and having a high number of psychosocial [β_a_ = -3.6 (95% CI: -7.1;-0.2)] and social area needs [β_a_ = -7.7 (95% CI: -13.4;-2.0)]. These explain 39.2% of the variance in men. Clinical diagnosis was not associated with MCS in either men or women (Table 5).

**Table 5:** Model of the mental component score of HRQOL by gender.

**WOMEN**		**Coefficient (IC95%)**	**sig.**	**Adjusted coefficient (IC95%)**	**sig.**
**Work Status**	Unemployed	0		0	
	Employed	11.4 (6.4:16.5)	0.00	6.1 (1.5:10.7)	0.01
**Number of friends**	0–3	0		0	
	4–6	6 (0.3:11.7)	0.04	4.2 (-0.6:8.9)	0.08
** **	≥7	10 (4.6:15.4)	0.00	6.6 (2.1:11.1)	0.01
**Social relationships**^**a**^	Worse	0		0	
	Improved or no change	13.5 (9:17.9)	0.00	8.6 (4.4:12.8)	0.00
**Type of needs**^**b**^	Health	-6.5 (-8.7:-4.4)	0.00	-3.9 (-6:-1.8)	0.00
**MEN**		**Coefficient (IC95%)**	**sig.**	**Adjusted coefficient (IC95%)**	**sig.**
**Living situation**	With someone	0		0	
	Alone	-7.5 (-14.4:-0.6)	0.03	-7.1 (-12.7:-1.4)	0.02
**Economic benefits**	Receives	0		0	
	Not receive benefits	12.6 (6.8:18.5)	0.00	8.5 (3.2:13.8)	0.00
**Social relationships**^**a**^	Worse	0		0	
	Improved or no change	10.8 (5.4:16.3)	0.00	6.8 (1.4:12.1)	0.01
**CAN-R**^**b**^	Total	-3 (-4.7:-1.4)	0.00	-3.6 (-7.1:-0.2)	0.04
**Type of needs**^**b**^	Social	-5.2 (-9.1:-1.3)	0.01	-7.7 (-13.4:-2)	0.01
	Health	-5.6 (-8.6:-2.6)	0.00	-8.1 (-12.6:-3.6)	0.00

IC95%: Confidence Interval of 95%

^a^ Social relationships and activities perceptions

^b.^ According the Camberwell Assessment of Need Questionnaire (CAN-R).

## Discussion

The main results of this study are: 1) the mean PCS of HRQOL in women with SMI is significantly lower than that of men with SMI, whereas in the case of MCS, there are no observed statistically significant differences; 2) however, there are differences in the factors associated with PCS and MCS of HRQOL between women and men with SMI.

Consistent with other studies, we have found that women with SMI present a statistically lower PCS of HRQOL than men with SMI[15–17]. For example, it has been reported that women with panic disorders have lower mean scores of HRQOL in the physical activity scale than men[41]. These results highlight that there are gender differences in general in our society and specifically, in people with mental health problems[42], both in psychiatric morbidity as well as in the pattern of behavior of different mental illnesses developed by men and women. The evidence indicates that the sociocultural factors that act through socially imposed roles and behavioral patterns are those that ultimately influence the way in which women and men display and cope with their psychological suffering. Over the last few decades, various studies have demonstrated that women often receive inferior and insensitive treatment by public institutions and by society in general, which either has no laws to prevent this behavior or fails to enforce them when it does[43].

However, unlike what has been reported in other studies[44], we did not find any significant statistical differences between women and men SMI with regard to MCS. For example, it has been reported that men with schizophrenia have worse MCS than women. This is explained by the fact that in the country under study, men have greater responsibility for supporting their families[44]. Nevertheless, this relationship was not observed in this study.

In addition, we found in our study that some factors are associated with HRQOL in both women and men with SMI. As seen in other studies, having a high number of psychosocial needs in the area of health is associated with a worse PCS and MCS of HRQOL in both men and women[31–33]. Furthermore, not having physical comorbidity is associated with better PCS in women and in men, as has been reported in other studies[20,27,28].

On the other hand, we found some factors associated with HRQOL in persons with SMI in one of the two genders but not in the other. One of the factors that we found associated with HRQOL in women with SMI but not in men is age. This is generally negatively associated with HRQOL[18,19], especially in PCS[20]. This difference could be explained by women generally having a longer life expectancy but with more chronic illnesses and with fewer years of good health and, as a result, poorer quality of life than men[45]. These inequalities can be attributed to a more precarious labor market for women and therefore, a lower socioeconomic position with a double work load of paid employment and unpaid work in the home[46].

Level of education is not always associated with HRQOL[25,47,48], but in our study we observed that men with a higher level of education have better HRQOL than those that do not. Specifically, this association has been observed in PCS[18,20]. However, being in work is associated with better HRQOL in both components[21–23,49] for women. Both factors are indicators of socioeconomic position and according to the differences observed in our results we can say that they affect women and men differently, which leads us to believe that there are gender inequalities in HRQOL for people with SMI. Being in work seems to imply economic independence, which occurs later in women than in men, and makes them feel more integrated in society and able to contribute value through their abilities. Employment is thus a key tool for personal development and realization and is associated with better HRQOL. Nevertheless, gender inequalities continue to exist in employment (like wage inequality), the home (unequal work load for domestic tasks and their role as informal caregivers) and the broader community (continued acceptation of higher levels of violence against women)[50]. However, in men, this role is assumed to be traditional and, together with greater employability, results in this factor not explaining their HRQOL. On the other hand, level of education is the socioeconomic factor that is associated with better HRQOL in men. This fact can be explained because level of education ends up determining employment status, which influences the possibility and type of job position.

Other indicators of socioeconomic position are income and economic benefits, which are associated with better HRQOL[23,24] in both women and men. However, this association has been observed in different components according to gender, had income of greater than 700 euros/month is positively associated in PCS in women and lacking economic benefits are positively associated in MCS in men. These results agree with a report by the OECD on how increasing participation of women in economic life reduces gender inequality in the workplace and provides benefits for all[51]. In addition, it has been shown that social class, specifically belonging to a lower social class, is a key determinants of poor mental health in the general population, in both women and men[52].

A greater number of friends is also positively associated with MCS of HRQOL[21,22,31] in women. It is shown that social relations act as a protective factor for health[53] through different aspects including: access to direct help in the case of the need for care; a dissuasive effect that exercises social control over the practice of risky activities; a greater level of social integration that leads to access to resources; and the feeling of belonging to a group[54]. On the other hand, living alone is negatively associated with MCS of HRQOL[25] in men but not in women. Even though it was not observed in this study, there is a broad consensus that living together as a couple has a positive and immediate effect on the health of both men and women in the middle and long term, although in lesser intensity in the case of women[55,56]. These benefits are explained by three factors: reduction of risky and unhealthy habits[57]; creation and maintenance of a social support network to which they can turn to in critical situations[55]; and increased material well-being resulting from economies of scale by combining resources and the specialization of tasks between the couple[56].

In terms of social functioning, the total number and different areas of psychosocial needs is associated with HRQOL, as has been observed in numerous studies[22,31–33]. In this study, it was primarily seen in men. The psychosocial needs in the area of services is associated with PCS. This area of the CAN-R responds to aspects of information, telephone, transport and benefits related to a physical dimension. Psychosocial needs in the social area of the CAN-R, which refer to aspects of companionship, intimate relationships and sexual expression related to the psychological dimension, are associated with MCS. The total number of psychosocial needs are also associated with MCS of HRQOL because a greater number of psychosocial needs, and thus problems in daily life, in these persons worsens the MCS scores of HRQOL.

Current self-perception of improvement or maintenance of social relationships and leisure activities is associated with MCS in women and men. Self-perception is a belief, and believing that social relationships and leisure activities, and therefore the social network status (previously mentioned as a protective factor determinant of health), have been maintained or improved is associated with MCS of HRQOL[53,54].

### Strengths and limitations

SMI groups different clinical diagnoses together that share a series of characteristics. This is both a strength because there are not many studies of patients with these clinical characteristics. At the same time, it involves a certain difficulty when comparing results with other studies because most of them work with populations with a single diagnosis, mostly schizophrenia, or with different groupings of diagnosis that do not strictly correspond with the definition of SMI employed in this study.

There is a lot of variability among HRQOL studies of people with SMI, mostly because of different definitions of the concept (HRQOL) itself and at the same time from the variety of measurement instruments used. This study, however, used a validated instrument, SF-36. Furthermore, a lack of evidence has been observed concerning explicative variables of HRQOL in people with SMI according to gender.

## Conclusions

To improve HRQOL in people living in the community with SMI, priority should be given to programs or interventions that address the control and management of symptoms and clinical needs and socioeconomic aspects like level of education, reentering the labor force, economic level, etc., as well as improved development of social networks. However, these interventions also require a focus on gender to address the differences observed in the factors associated with HRQOL in women and men.

## References

[1] Generalitat de Catalunya. Departament de Salut. Proposta de Pla de salut de Catalunya 2016–2020. Barcelona; 2016.

[2] Grupo de la OMS sobre la calidad de vida. ¿Qué calidad de vida? La gente y la salud. Foro Mundial de la Salud. 1996.

[3] Urzúa A. Calidad de vida relacionada con la salud: Elementos conceptuales. Revista médica de Chile. 2010;138: 358–365. 10.4067/S0034-98872010000300017 20556342

[4] Prigent A, Auraaen A, Kamendje-Tchokobou B, Durand-Zaleski I, Chevreul K. Health-related quality of life and utility scores in people with mental disorders: A comparison with the non-mentally ill general population. International Journal of Environmental Research and Public Health. 2014;11: 2807–2817. 10.3390/ijerph110302804 24608903 PMC3987005

[5] Green MJ, Benzeval M. The development of socioeconomic inequalities in anxiety and depression symptoms over the lifecourse. Social Psychiatry and Psychiatric Epidemiology. 2013;48: 1951–1961. 10.1007/s00127-013-0720-0 23732706 PMC3830746

[6] Becker M, Diamond R. Nuevos avances en la medición de la calidad de vida en la esquizofrenia In: Katsching H, Freeman H, Sartorius N, editors. Calidad de vida en los trastornos mentales. Barcelona: Masson; 2000 pp. 115–128.

[7] Awad G. Calidad de vida en esquizofrenia. Conceptos y medidas para la práctica clínica. Revista Psiquiatria Uruguay. 2008;72: 121–129.

[8] Bobes J, González MP, Bascarán MT, Arango C, Sáiz PA, Bousoño M. Calidad de vida y discapacidad en pacientes con trastorno obsesivo-compulsivo. Eur Psychiatry Ed Esp. 2001;16: 436–442. 10.1016/s0924-9338(01)00571-5 11418275

[9] Organización Mundial de la Salud. Salud mental: fortalecer nuestra respuesta [Internet]. Ginebra: World Health Organization; 2016 [cited 2 Apr 2016]. Available: http://www.who.int/mediacentre/factsheets/fs220/es/

[10] Asociación Española de Neuropsiquiatría. Rehabilitación psicosocial del trastorno mental severo Situación actual y recomendaciones. Madrid; 2002.

[11] Gumà J, Treviño R. Posición en el hogar y género. Desigualdades en la calidad de vida relacionada con la salud entre la población adulta en España. Revista Internacional de Sociología. 2015;73: 1–17. 10.3989/2013.03.04

[12] Vives-Cases C, Eriksson M, Goicolea I, Öhman A. Gender and health inequalities: intersections with other relevant axes of oppression. Global health action. 2015; 10.3402/gha.v8.30292 28156837 PMC4666892

[13] Grundy E. Gender and social inequities in health—a public health issue. Wamala SP, Lynch J, editors. 2002.

[14] Holloway F, Carson J. Quality of life in severe mental illness. Int Rev Psychiatry. 2002;14: 175–184.

[15] Baladón L, Rubio-Valera M, Serrano-Blanco A, Palao DJ, Fernández A. Gender differences in the impact of mental disorders and chronic physical conditions on health-related quality of life among non-demented primary care elderly patients. Quality of Life Research. 2016;25: 1461–1474. 10.1007/s11136-015-1182-5 26590837

[16] Galuppi A, Turola MC, Nanni MG, Mazzoni P, Grassi L. Schizophrenia and quality of life: how important are symptoms and functioning? International journal of mental health systems. 2010;4: 1–8. 10.1186/1752-4458-4-1 21143871 PMC3016370

[17] Hofer A, Baumgartner S, Edlinger M, Hummer M, Kemmler G, Rettenbacher MA, et al Patient outcomes in schizophrenia I: correlates with sociodemographic variables, psychopathology, and side effects. European psychiatry: the journal of the Association of European Psychiatrists. 2005;20: 386–94. 10.1016/j.eurpsy.2005.02.005 16171653

[18] Brissos S, Balanzá-Martinez V, Dias VV, Carita AI, Figueira ML. Is personal and social functioning associated with subjective quality of life in schizophrenia patients living in the community? European Archives of Psychiatry and Clinical Neuroscience. 2011;261: 509–517. 10.1007/s00406-011-0200-z 21384166

[19] Montemagni C, Castagna F, Crivelli B, De Marzi G, Frieri T, Macrì A, et al Relative contributions of negative symptoms, insight, and coping strategies to quality of life in stable schizophrenia. Psychiatry Research. 2014;220: 102–111. 10.1016/j.psychres.2014.07.019 25128248

[20] Beard C, Weisberg RB, Keller MB. Health-related Quality of Life across the anxiety disorders: Findings from a sample of primary care patients. Journal of Anxiety Disorders. 2010;24: 559–564. 10.1016/j.janxdis.2010.03.015 20418054 PMC2876232

[21] Pitkänen A, Hätönen H, Kuosmanen L, Välimäki M. Individual quality of life of people with severe mental disorders. Journal of Psychiatric and Mental Health Nursing. 2009;16: 3–9. 10.1111/j.1365-2850.2008.01308.x 19192080

[22] Hansson L. Determinants of quality of life in people with severe mental illness. Acta Psychiatrica Scandinavica. 2006;113: 46–50. 10.1111/j.1600-0447.2005.00717.x 16445482

[23] Hsiao CY, Hsieh MH, Tseng CJ, Chien SH, Chang CC. Quality of life of individuals with schizophrenia living in the community: Relationship to socio-demographic, clinical and psychosocial characteristics. Journal of Clinical Nursing. 2012;21: 2367–2376. 10.1111/j.1365-2702.2012.04067.x 22788567

[24] Bengtsson-Tops A, Hansson L, Sandlund M, Bjarnason O, Korkeila J, Merinder L, et al Subjective versus interviewer assessment of global quality of life among persons with schizophrenia living in the community: a Nordic multicentre study. Quality of life research. 2005;14: 221–9. 10.1007/s11136-004-3926-5 15789956

[25] Heider D, Angermeyer MC, Winkler I, Schomerus G, Bebbington PE, Brugha T, et al A prospective study of Quality of life in schizophrenia in three European countries. Schizophrenia research. 2007;93: 194–202. 10.1016/j.schres.2007.03.005 17466491

[26] Corrigan PW, Buican B. The construct validity of subjective quality of life for the severely mentally ill. J Nerv Ment Dis. 1995;183: 281–85. 10.1097/00005053-199505000-00001 7745380

[27] Barnes AL, Murphy ME, Fowler CA, Rempfer M V. Health-related quality of life and overall life satisfaction in people with serious mental illness. Schizophrenia research and treatment. 2012;2012 10.1155/2012/245103 23213525 PMC3507053

[28] Neil AO, Stevenson CE, Williams ED, Mortimer D, Oldenburg B, Sanderson K. The health-related quality of life burden of co-morbid cardiovascular disease and major depressive disorder in Australia: findings from a population-based, cross-sectional study. Qual Life Res. 2013;22: 37–44. 10.1007/s11136-012-0128-4 22323040 PMC3548108

[29] Saarni SI, Viertiö S, Perälä J, Koskinen S, Lönnqvist J, Suvisaari J. Quality of life of people with schizophrenia, bipolar disorder and other psychotic disorders. The British journal of psychiatry: the journal of mental science. 2010;197: 386–94. 10.1192/bjp.bp.109.076489 21037216

[30] Lasalvia A, Ruggeri M, Santolini N. Subjective quality of life: its relationship with clinician-rated and patient-rated psychopathology. The South-Verona Outcome Project 6. Psychother Psychosom. 2002;71: 275–84. 10.1159/000064809 12207108

[31] Lambri M, Chakraborty A, Leavey G, King M. Quality of life and unmet need in people with psychosis in the London Borough of Haringey, UK. The Scientific World Journal. 2012;2012 10.1100/2012/836067 23213300 PMC3506896

[32] Wiersma D, van Busschbach J. Are needs and satisfaction of care associated with quality of life? An epidemiological survey among the severely mentally ill in the Netherlands. Eur Arch Psychiatry Clin Neurosci. 2001;251: 239–246. 10.1007/s004060170033 11829211

[33] Bengtsson-Tops A, Hansson L. Clinical and social needs of schizophrenic outpatients living in the community: the relationship between needs and subjective quality of life. Social psychiatry and psychiatric epidemiology. 1999;34: 513–8. 10.1007/s001270050169 10591810

[34] Ware JE, Sherbourne CD. The MOS 36-item short-form health survey (SF-36). I. Conceptual framework and item selection. Medical care. 1992;30: 473–83. 1593914

[35] Alonso J, Antó J, Prieto L. La versión española del SF-36 Health Survey (Cuestionario de Salud SF-36): un instrumento para la medida de los resultados clínicos. Medicina Clínica. 1995;104: 771–776. 7783470

[36] Phelan M, Slade M, Thornicroft G, Dunn G, Holloway F, Wykes T, et al The Camberwell Assessment of Need: the validity and reliability of an instrument to assess the needs of people with severe mental illness. The British journal of psychiatry: the journal of mental science. 1995;167: 589–595. 8564313 10.1192/bjp.167.5.589

[37] Rosales Varo C, Torres González F, Luna Del Castillo J, Jiménez Estévez J, Martínez Montes G. Reliability of the Spanish version of the Camberwell Assessment of Needs (CAN) (Spanish version of CAN Reliability Study). Actas españolas de psiquiatría. 2002;30: 99–104. 12028942

[38] Meijer K, Schene A, Koeter M, Knudsen HC, Becker T, Thornicroft G, et al Needs for care of patients with schizophrenia and the consequences for their informal caregivers. Social Psychiatry and Psychiatric Epidemiology. 2004;39: 251–258. 10.1007/s00127-004-0737-5 15085325

[39] McCrone P, Leese M, Thornicroft G, Schene AH, Knudsen HC, Vázquez-Barquero JL, et al Reliability of the Camberwell Assessment of Needs–European version. EPSILON Study 6. Br J Psychiatry. 2000;177: 34–40. 10.1192/bjp.177.39.s34 10945076

[40] Kunkel SR, Atchley RC. Why gender matters: being female is not the same as not being male. American journal of preventive medicine. 1996;12: 294–6. 8909635

[41] Kim JE, Song IH, Lee S. Gender Differences of Stressful Life Events, Coping Style, Symptom Severity, and Health-Related Quality of Life in Patients With Panic Disorder. The Journal of Nervous and Mental Disease. 2017;205: 714–719. 10.1097/NMD.0000000000000696 28609311

[42] Borrell C, Artazcoz L, Gil-González D, Pérez K, Pérez G, Vives-Cases C, et al Determinants of perceived sexism and their role on the association of sexism with mental health. Women and Health. 2011;51: 583–603. 10.1080/03630242.2011.608416 21973112

[43] Turshen M. The Impact of Sexism on Women’s Health and Health Care. J Public Health Policy. 1993;14: 164–173. 8408607

[44] Li Y, Hou C-L, Ma X-R, Zhong B-L, Zang Y, Jia F-J, et al Quality of life in Chinese patients with schizophrenia treated in primary care. Psychiatry Research. 2017;254: 80–84. 10.1016/j.psychres.2017.04.049 28456026

[45] Espelt A, Font-Ribera L, Rodriguez-Sanz M, Artazcoz L, Ferrando J, Plaza A, et al Disability Among Older People in a Southern European City in 2006: Trends in Gender and Socioeconomic Inequalities. Journal of Women’s Health. 2010;19: 927–933. 10.1089/jwh.2009.1608 20438304

[46] Dahlin J, Härkönen J. Cross-national differences in the gender gap in subjective health in Europe: Does country-level gender equality matter? Social Science and Medicine. 2013;98: 24–28. 10.1016/j.socscimed.2013.08.028 24331878

[47] Fervaha G, Agid O, Takeuchi H, Foussias G, Remington G. Clinical determinants of life satisfaction in chronic schizophrenia: Data from the CATIE study. Schizophrenia Research. 2013;151: 203–208. 10.1016/j.schres.2013.10.021 24183751

[48] Kunikata H, Mino Y, Nakajima K. Quality of life of schizophrenic patients living in the community: The relationships with personal characteristics, objective indicators and self-esteem. Psychiatry and Clinical Neurosciences. 2005;59: 163–169. 10.1111/j.1440-1819.2005.01352.x 15823162

[49] Nordt C, Müller B, Rössler W, Lauber C. Predictors and course of vocational status, income, and quality of life in people with severe mental illness: A naturalistic study. Social Science and Medicine. 2007;65: 1420–1429. 10.1016/j.socscimed.2007.05.024 17583402

[50] WHO. Regional Office for Europe. Beyond the mortality advantage Investigating women’s health in Europe. Copenhagen: World Health Organization; 2015.

[51] OECD. In It Together. Why Less Inequality Benefi ts All. Paris: OECD Publishing; 2015 10.1787/9789264235120-en

[52] Arias-de la Torre J, Molina AJ, Fernández-Villa T, Artazcoz L, Martín V. Mental health, family roles and employment status inside and outside the household in Spain. Gaceta Sanitaria. 2017; 10.1016/j.gaceta.2017.11.005 29452752

[53] Fleury M-J, Grenier G, Bamvita J-M, Tremblay J, Schmitz N, Caron J. Predictors of quality of life in a longitudinal study of users with severe mental disorders. Health and quality of life outcomes. 2013;11: 1–12. 10.1186/1477-7525-11-1 23758682 PMC3681595

[54] Berkman LF, Glass T, Brissette I, Seeman TE. From social integration to health: Durkheim in the new millennium. Social Science & Medicine. 2000;51: 843–857. 10972429 10.1016/s0277-9536(00)00065-4

[55] Waite LJ. Does Marriage Matter? Demography. 1995;32: 483–507. 10.2307/2061670 8925942

[56] Lillard LA, Panis CWA. Marital status and mortality: the role of health. Demography. 1996;33: 313–327. 10.2307/2061764 8875065

[57] Lillard LA, Waite LJ. Til death do us part: marital disruption and mortality. American Journal of Sociology. 1995;100: 1131–1156. 10.1086/230634

