# Supplementary material for: Gender differences in health-related quality of life in people with severe mental illness
Source: PLoS One. 2020 Feb 21;15(2):e0229236. doi: 10.1371/journal.pone.0229236 (PMC7034846; doi:10.1371/journal.pone.0229236)
Supplement: Attachment — Submitted filename: Colillas-Malet.Response to Reviewers.docx [file pone.0229236.s001.docx]

**Title:** **Gender Inequality in Health-Related Quality of Life in people with Severe Mental Illness**

**Submission number:** PONE-D-19-20978

Dear Editor,

In first place we want to thank you for the new reviewers’ comments. Below you can find the answers to each of the comments and all changes made are highlighted in track changes mode activated in the manuscript:

**REVIEWER 1**

COMMENT 1:

*Please add in more details for the study process including simple random sampling method used in the current study, like how the sample frame was determined, who approached the patients at the first place, how to avoid potential coercion, any inconvenience fees, etc. The current description is very vague.*

AUTHORS ANSWER: It has been rewritten the sample selection section adding more details for the study process: *There was a total of 783 people who met the inclusion criteria (see line 63).* *The sample was selected by simple random sampling and* t*he size was determined based on the known size of the population that met inclusion criteria, with a 5% error and a 3% accuracy. The theoretical sample was 258 people diagnosed with SMI who carried out ambulatory follow-up at mental health center.* (see line 70-73)

COMMENT 2:

*Were the data collected through interview or self-administered questionnaire? In the sample selection authors mentioned through interview; however, in the variables authors then mentioned 'it's a self-administer questionnaire...' This is confusing, please make it clear.*

AUTHORS ANSWER: It has been deleted the word “self-administred”, see line 83, because the questionnaire finally was administered by a trained interviewer.

COMMENT 3:

*For the CAN-R - 1) please spell in full in when it first appears in the manuscript, and put the abbreviation in the brackets; 2) is the tool validated among people with severe mental illness in Spain? also how about the domains? In the citing reference, their study sample comprised individuals with schizophrenia from 5 different European countries, which is quite different from the current study sample?*

AUTHORS ANSWER: 1)It has been added the complete name of the CAN-R, Camberwell Assessment of Need, see line 97; 2) The tool has been used is validated among people with schizophrenia, considered a long-acting psychotic disorder such as severe mental illness; The 22 domains can also be grouped in five dimensions (CAN manual, Research version 3.0-E):

A. Basic (3 domains): accommodation, food and daytime activities

B. Health (7 domains): physical health, psychotic symptoms, psychological distress, safety to self, safety to others, alcohol and drugs

C. Social (3 domains): company, intimate relationships and sexual expression

D. Functioning (5 domains): looking after the home, self-care, childcare, education and money

E. Services (4 domains): information, telephone, transport and benefits.

The CAN-EU has good inter-rater and test-retest reliability, the authors has been added a new reference, number 39 (see line 106).

COMMENT 4:

*Variables such as 'social consequences', 'possibilities for the future', 'help in the future' and 'economic benefits' were very confusing, please give more details.*

AUTHORS ANSWER: It has been explained more details about these variables*: as for example, if the SMI had had consequences on their social life and leisure activities, if these social consequences had improved or worsened their social relationships, if they had considered what possibilities would be in the future and if they thought that would need help.* (see lines 107-110)

COMMENT 5:

*How was 'num. of need CAN' determined? This is unclear throughout the whole paper.*

AUTHORS ANSWER: It has been modified how was number of needs CAN determined: *All the areas are scored from 0 to 1 with: 0, no problem; 1, moderate or serious problem due to help given.*  (see lines 103-104)

COMMENT 6:

*For work status - shouldn't people with paid sick leave also fall under employed?*

AUTHORS ANSWER: The authors divided this variable in two categories (employed/unemployed) because we considered if the participant was working in the moment of the interview or not.

COMMENT 7:

*Why was 'number of friends' not a continuous variable?*

AUTHORS ANSWER: This is a categorical variable because from the beginning it was posed as such. In any case, this consideration will be taken in account for upcoming investigations.

COMMENT 8:

*For social relationships - compared to when?*

AUTHORS ANSWER: It has been explained more details about this variable: *as for example, if the SMI had had consequences on their social life and leisure activities, if these social consequences had improved or worsened their social relationships, if they had considered what possibilities would be in the future and if they thought that would need help.* (see line 109)

COMMENT 9:

*I'm not quite sure how the authors conducted the regression analyses. In the current paper the description was not clear. I need more details to tell whether the statistical analyses were done properly.*

AUTHORS ANSWER: The authors has been explained more details of the statistical analyses: *The variables that presented a p<0.1 in the bivariate model were included in the multivariate models. In addition, the colineality of these variables was studied.* (see lines 121-123)

**REVIEWER 2**

COMMENT 1:

*In line 21, directly writing the acronym “SMI”, it is important to explain what this means.*

AUTHORS ANSWER: It has been added the abbreviation in line 4, that is in the first place that this concept appears.

COMMENT 2:

*In paragraph 29 through 31, the sentence “There are many people with mental health problems in the community and their HRQOL is especially important to consider. They often live in more disadvantaged conditions compared to the rest of the general population.” You have to put the references and explain why this is what you describe.*

AUTHORS ANSWER: It has been added references in lines 29-31.

COMMENT 3:

*The same, in the sentence of lines 31-32 "They often live in more disadvantaged conditions compared to the rest of the general population". Must have references.*

AUTHORS ANSWER: It has been added references in lines 31-32.

COMMENT 4:

*At line 79, “Spanish version [32] of the Medical Outcome Study Short Form 36 (SF-36)” I suggest writing the name of the questionnaire in Spanish, too.*

AUTHORS ANSWER: It has been written the name of the questionnaire in Spanish: *Cuestionario de Salud SF-36.* (see line 83)

COMMENT 5:

*Similarly, line 94, written “the Spanish version of the CAN-R [34]”, has to describe what it means as well as in English and Spanish.*

AUTHORS ANSWER: It has been written the name of the questionnaire in Spanish: *Cuestionario Camberwell para la evaluación de necesidades.* (see line 98)

We hope that the changes done in the manuscript and the responses given to the comments are of your compliance, making possible the acceptance of the manuscript for being published on PlosOne.

We thank you in advance your evaluation.

Sincerely,

Authors.
