# Supplementary material for: Gender differences in health-related quality of life in people with severe mental illness
Source: PLoS One. 2020 Feb 21;15(2):e0229236. doi: 10.1371/journal.pone.0229236 (PMC7034846; doi:10.1371/journal.pone.0229236)
Supplement: Attachment — Submitted filename: Colillas-Malet.Response to Reviewers.docx [file pone.0229236.s002.docx]

**Title:** **Gender Inequality in Health-Related Quality of Life in people with Severe Mental Illness**

**Submission number:** PONE-D-19-20978R1

Dear Editor,

In first place we want to thank you for the new reviewer’ comments. Below you can find the answers to each of the comments and all changes made are highlighted in track changes mode activated in the manuscript:

**REVIEWER**

COMMENT 1:

*Regarding authors' responses, my comment 2 - author suggested that they had clarified self-administered in line 83, which I didn't see, please clarify.*

AUTHORS ANSWER: It has been rewritten the sample selection section adding more details for the study process: *The participants were recruited by telephone, which also served to specify the date and time for the interview. The interview consisted of a 30-minute evaluation where a trained interviewer filled out the various questionnaires.* (see lines 77-79)

COMMENT 2:

*My comment 6 - I'm not convinced by the current explanation. In our local context, normally individuals would only take 1 day sick leave (paid), unless they had severe conditions and have to be hospitalized. In this case, it makes more sense to be categorized into employed group. Please provide more specified reason why paid sick leave should be categorized into unemployed group if there are any cultural differences (and add the details into the revised manuscript).*

AUTHORS ANSWER: The authors categorized this variable thus because, as argued in the discussion section: *being in work makes women feel more integrated in society and able to contribute value through their abilities. Employment is thus a key tool for personal development and realization and is associated with better HRQOL*. Therefore, beyond being an indicator of socioeconomic position also is an indicator for personal development and realization and this aspect was of particular interest to us we had other variables that reflect aspects of socioeconomic position.

COMMENT 3:

*The way authors presenting the regression results is confusing - please use different ways for associations with different directions. Take PCS among women for example, better PCS is POSITIVELY associated with age, but NEGATIVELY associated higher number of health related needed.*

AUTHORS ANSWER: It has been rewritten this part in the paragraph of PCS: *The PCS for HRQOL shows lower scores in women with SMI than men with SMI (*$\bar{x}$*_women_=44.6 and* $\bar{x}$*_men_=49.0; p=0.00) (Table 1). The women presenting better PCS were: unmarried,* $\bar{x}$*=46.3; had higher levels of education/college educated,* $\bar{x}$*=51.7; in work,* $\bar{x}$*=52.3; had bipolar disorder,* $\bar{x}$*=49.4; without physical comorbidity,* $\bar{x}$*=49.9; and had seen their social relationships and leisure activities as having improved or not changed,* $\bar{x}$*=49.0. However, age (r=-0.3) and had a lower number of psychosocial needs (r=-0.5) was negatively associated with PCS in women. On the other hand, men, like women, who presented better PCS were: in work,* $\bar{x}$*=52.2; without physical comorbidity,* $\bar{x}$*=51.6; and had seen their social relationships and leisure activities as having improved or not changed,* $\bar{x}$*=52.4. However, age (r=-0.2) and had a lower number of psychosocial needs (r=-0.5) also was negatively associated with PCS in men*. (see lines 141-150)

Also, it has been rewritten this part in the paragraph of MCS*: Men and women had similar MCS scores for HRQOL (*$\bar{x}$*_women_=36.4 and* $\bar{x}$*_men_=37.5) (Table 1). The women who had better MCS were those who: had secondary education,* $\bar{x}$*=40.9; were in work,* $\bar{x}$*=44.9; had more than 7 friends,* $\bar{x}$*=41.4; had bipolar disorder,* $\bar{x}$*=41.1; and those that perceived that their social relationships and leisure activities had improved or not changed,* $\bar{x}$*=43.9. However, had a lower number of psychosocial needs (r=-0.5) was negatively associated with MCS in women. For their part, men who presented better MCS, like their female counterparts, were: in work,* $\bar{x}$*=45.9; and had more than 7 friends,* $\bar{x}$*=42.7. In addition, unlike women, men living with someone presented better MCS,* $\bar{x}$*=39.0. However, had a lower number of psychosocial needs (r=-0.4) also was negatively associated with MCS in men.* (see lines 162-170)

COMMENT 4:

*Results on regressions - please list out the reference group if there are three or more categories for a variable (for 2 categories, it is also recommended to list out the reference group, it's more clear).*

AUTHORS ANSWER: It have added the reference categories to the categorical variables in tables 3 and 5.

COMMENT 5:

*In discussion, line 205-106 - according to the regression results, i thought co-morbidity is only significant among PCS, not among all HRQOL, right? Pls don't over interpret your findings.*

AUTHORS ANSWER: It has been rewritten this sentence because the comorbidity has been only significant among PCS: *Furthermore, not having physical comorbidity is associated with better PCS in women and in men, as has been reported in other studies.* (see lines 206-207).

COMMENT 6:

*In discussion line 210-212 - Are you sure life expectancy is a good explanation? according to your descriptive statistics, I don't think the age range is huge that can cause such gender differences.*

AUTHORS ANSWER: In the table 1 of the result it can be observed that women have a significantly higher mean age than men ($\bar{x}$_women_=44.9 years and $\bar{x}$_men_=41.3 years; p=0.01), and this factor together with the double work load, we considered can be explain this differences by gender.

COMMENT 7:

*In discussion line 233-237 - for income, isn't it only significant among MCS women? Where are these 'PCS in women' and 'MCS in men' come from?*

AUTHORS ANSWER: The authors are revised and rewritten this paragraph: *Other indicators of socioeconomic position are income and economic benefits, which are associated with better HRQOL[23,24] in both women and men. However, this association has been observed in different components according to gender, had income of greater than 700 euros/month is positively associated in PCS in women and lacking economic benefits are positively associated in MCS in men. These results agree with a report by the OECD on how increasing participation of women in economic life reduces gender inequality in the workplace and provides benefits for all[51]. In addition, it has been shown that social class, specifically belonging to a lower social class, is a key determinants of poor mental health in the general population, in both women and men[52].* (see lines 234-242)

COMMENT 8:

*After reading the whole article, I didn't see any gender inequity evidences other than men have better PCS scores, and this was only from a non-adjusted comparison. All other findings were about gender differences. In this case I think the manuscript title should be revised to 'gender differences ...'*

AUTHORS ANSWER: The authors are revised and rewritten the title: *Gender Differences in Health-Related Quality of Life in people with Severe Mental Illness. (see lines 1-2)*

We hope that the changes done in the manuscript and the responses given to the comments are of your compliance, making possible the acceptance of the manuscript for being published on PlosOne.

We thank you in advance your evaluation.

Sincerely,

Authors.
